# Turning a pathogen protein into a therapeutic tool for sepsis

**DOI:** 10.15252/emmm.202013589

**Published:** 2020-12-17

**Authors:** Tineke Vanderhaeghen, Charlotte Wallaeys, Claude Libert

**Affiliations:** ^1^ Center for Inflammation Research VIB Ghent Belgium; ^2^ Department of Biomedical Molecular Biology Ghent University Ghent Belgium

**Keywords:** Microbiology, Virology & Host Pathogen Interaction

## Abstract

Sepsis causes unacceptably high amounts of deaths worldwide. It is a huge unmet medical need, and new therapeutic interventions for sepsis and septic shock are urgently needed. By studying the mechanism by which a bacterial protein undermines the inflammatory function of macrophages, Kim *et al*, in the last issue of *EMBO Molecular Medicine*, have developed a new therapeutic protein drug, which appears to have very promising protective activities in a well‐validated and aggressive polymicrobial sepsis model in mice. The chimeric protein is thought to limit macrophage inflammation while activating phagocytosis, and so, it hits two macrophage pathways at once.

## Graphical abstract

T. Vanderhaeghen, C. Wallaeys and C. Libert discuss the identification of a new therapeutic protein drug against sepsis as reported by CS. Yang and colleagues, in the last issue of *EMBO Mol Med*.

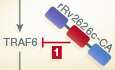

According to a recent paper, sepsis yearly hits up to 49 million people, killing 11 million of them (Rudd *et al*, 2020). This means that almost one in five deaths on the planet are due to sepsis and that, on a global scale, the risk of dying when diagnosed with sepsis is 22%. These staggering numbers underscore the 2017 call of WHO to consider sepsis as a top‐priority unmet medical need (Reinhart *et al*, 2017). Despite 78% of sepsis patients recover, many of them retain life‐long disabilities, *e.g*., cognitive problems and depression. Resuscitation, organ function support, antibiotics, and infection control form the cornerstones of today’s treatment (Van Der Poll *et al*, 2017). The need for innovative therapeutics, which are applicable in the most remote corners of the planet, is urgent and high.

The majority of sepsis cases results from bacterial infections. Only about a dozen infectious bacteria cover most of the cases, and considerable overlap exists between different sepsis forms, e.g., between peritonitis and pneumonia. Bacteria causing sepsis include Gram‐negative and Gram‐positive bacteria, mostly aerobics (Van Der Poll *et al*, 2017). The generation of sepsis‐preventing strategies, based on vaccination, has proven to be difficult, but not impossible. For example, several vaccines against *Streptococcus pneumoniae* are commercially available (Kaplonek *et al*, 2018). Besides, the development of therapeutic, inhibitory monoclonal antibodies directed against bacterial antigens is gaining interest. These strategies are directed toward the support of the immune system of the (potentially) infected patient.

The reason why the immune system needs help to overcome sepsis may lay in the relatively slow mode of action of the adaptive immune response. However, sepsis is typically an acute and severe infection, *e.g*., starting from an intestinal perforation (Van Wyngene *et al*, 2018). Given this acuteness, it is more up to the innate immune response to overcome the first days of sepsis. It is generally believed that macrophages and neutrophils are first in line to detect and clear the infection. They develop an acute inflammatory response, are attracted to the site of infection, and will kill invaders via the production of toxins (*e.g*., defensins, reactive oxygen intermediates) and phagocytosis. Despite these mechanisms are efficient and fast, they are associated with collateral damage to host cells and tissues, which may prove to be fatal (Venet & Monneret, 2018). However, the development of drugs that guide this innate immune response through this mine field, while remaining focused and efficient, would be beneficial. This is the merit of the paper of Kim *et al*, in the last issue of *EMBO Molecular Medicine* (Kim *et al*, 2020).

The authors focused their attention on a well‐known protein produced by *Mycobacterium tuberculosis*, the pathogenic bacteria causing tuberculosis. This protein, the 16 kDa big Rv2626c, has been studied extensively because it is known to have a strong impact on host macrophages (Bashir *et al*, 2010). Exclusively in the mouse model, Kim *et al* show that the protein, when presented to macrophages, enters the cells via a Toll‐like receptor (TLR)2‐dependent pathway. It is slightly inflammatory, as it induces some inflammatory cytokines. However, Rv2626c is strongly reducing the inflammation induced by lipopolysaccharides (LPS), which are cell wall components of Gram‐negative bacteria, which activate macrophages via the TLR4 route. Once in the cells, Rv2626c was found to interact with several interesting proteins, including Ripk1 (receptor‐interacting serine–threonine kinase 1) and TRAF6 (TNF receptor‐associated factor 6). The interaction of Rv2626c with TRAF6 is the main focus of the paper. The interaction of Rv2626c and Ripk1 could also be of great interest, since Ripk1 is essential in progression of inflammation and cell death. TRAF6 is known as a scaffold protein, which integrates several signaling molecules and allows them to interact in complexes. TRAF6 itself becomes labeled with ubiquitin chains, which are coupled in a K63 linear way (meaning that ubiquitin monomers are coupled to the lysin on position 63 of the previous ubiquitin monomer). This linear K63‐linked polyubiquitination is essential for the activation of the pro‐inflammatory transcription factor NF‐κB. Kim *et al* show that Rv2626c inhibits the formation of this K63 ubiquitin chain on TRAF6. Since Ripk1 is also K63‐ubiquitinated, the impact of Rv2626c on this chain would be great to know. By a number of elegant experiments, they also pinpoint a stretch of 9 amino acids of the Rv2626c protein, which are key in the binding and muting of TRAF6.

Kim *et al* developed a new recombinant chimeric protein by first linking 5 copies of the 9 amino acids of Rv2626c that bind to TRAF6 to a cell‐penetrating peptide on the N‐terminal side via a short linker. This was then fused to 10 copies of the Tuftsin motif of 4 amino acids (TKPR) on the C‐terminal side, leading to the generation of a protein of 101 amino acids, called rRv2626c‐CA. This protein penetrates cells, inhibits TRAF6, and is suspected to have the phagocytosis activating effects of the Tuftsin motif.

When added to macrophages, rRv2626c‐CA shows TRAF6 inhibition and anti‐inflammatory effects, when cells are stimulated with LPS (Fig 1). Interestingly, this protein has very robust protective effects in a polymicrobial sepsis mouse model, namely the cecal ligation and puncture (CLP) model. This highly validated model causes a lethal septic peritonitis, very similar as in humans (Dejager *et al*, 2011). The attractive aspects of this therapy are its quick establishment (by 4 injections at 0, 6, 12 and 24 h after CLP initiation), its dose responsiveness, and turning an LD_90_ sepsis into an LD_10_, leading to a significant protection in the CLP model. When the therapeutic protein is labeled, it accumulates in several tissues, almost exclusively in macrophages, which, by means of the protein, have undergone a clear shift toward the M2 type of macrophages.

**Figure 1:**
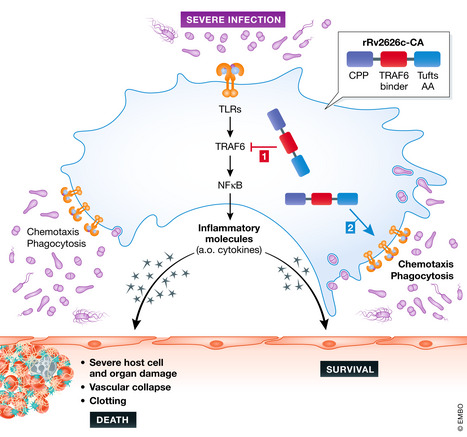
Sepsis is associated with bacteria, which stimulate phagocytes via several different surface molecules, including Toll‐like receptors These receptors will activate inflammatory transcription factors, the best characterized being NF‐__B. TRAF6 is a central scaffold regulatory molecule. Inflammation, which is aimed to stimulate the macrophages to kill the bacteria, move toward them, and engulf them by phagocytosis, but it causes also collateral damage to host cells and tissues, causing death of the host. The rR2626c‐CA protein can be sneaked into cells thanks to a cell permeability peptide, dampens TRAF6 by a TRAF6‐binding part, thereby limiting inflammation, but at the same time activates chemotaxis and phagocytosis, thanks to a Tufts peptide repeat.

rRv2626c‐CA has a significant therapeutic impact in the CLP model. However, before moving into human sepsis, there is more work to be done. It will be essential to show safety of the therapy, but also to show therapeutic effects in other mouse models of sepsis, predominantly in pneumonia models and urogenital and brain sepsis models. Also, therapeutic effects in larger pre‐clinical models, such as pigs, will be mandatory. Furthermore, the real mechanism underlying the therapy is not clear. The macrophages of the protected animals indeed express some typical markers associated with phagocytosis, but the authors have not shown that rRv2626c‐CA stimulates phagocytosis of particles or bacteria. Also, the reason why the protein appears to hit macrophages preferentially is not clear. These and other studies, e.g., using TRAF6‐deficient mice, would be most welcome to study this mechanism in more depth.
